# Special communication: China’s first historic efforts to develop a tobacco control advocacy workforce via schools of public health

**DOI:** 10.1136/tc.2009.031815

**Published:** 2009-07-20

**Authors:** T Yang, X Yang, Q Lv, Q Zhao, X Ke

**Affiliations:** 1 Center for Tobacco Control Research, Zhejiang University School of Medicine, China; 2 Department of Sociology, Zhejiang University, China

## Abstract

This paper provides an overview of a recent 18-month project which set out for the first time to introduce training on tobacco control into the curricula of public health courses in Chinese universities. The aim was to produce graduates with appropriate knowledge and skills to be effective in advocating for policies that could lead to the reduction of tobacco use. Results from this initial project involving seven universities have been encouraging and the new curriculum is to be implemented, with some changes, on a wider scale throughout China. Each of the universities also successfully introduced a smoke-free campus policy and the aim is to extend this policy.

In China tobacco use has reached epidemic proportions.^1^ China leads the world both in consumption of tobacco and in smoking-related deaths. Large-scale epidemiological studies have shown that smoking was responsible for approximately one million deaths annually in China during the 1990s.^2^ This number is projected to reach two million per year by 2025 and increase to three million by 2050. Approximately 100 million Chinese will die of smoking-related causes over the next 50 years if the current smoking rate continues.^3^ ^4^

To counter the spread of tobacco use worldwide, the World Health Organization established the Framework Convention on Tobacco Control (FCTC) in 1999. This was fully endorsed by member states (including China) on 21 May 2003 and was ratified by the Chinese National People’s Congress in 2005. Yet implementation of tobacco control activities associated with the FCTC initially progressed very slowly in China. One reason for this was the lack of skilled personnel who could act as advocates to promote tobacco control activities and policies.

Currently 68 universities in China have either a school or department of public health. About 5000 students graduate annually from these institutions with a degree in public health. Yet the absence of education on tobacco control in their curricula has meant that many of these graduates do not have appropriate knowledge and skills to be effective in advocating policies that can lead to the reduction of tobacco use. This deficiency in training is caused by the lack of institutional resources to support faculty members in teaching tobacco control. The development of an infrastructure for training is urgently needed in China in order to build capacity for tobacco control.

## Project profile

The project described below was an 18-month training trial to build capacity for tobacco control among new graduates in the Chinese public health workforce. The aim was to create a training infrastructure in some of the nation’s schools of public health and/or the departments of public health in schools of medicine. The training programme was designed to fit well with Chinese culture, and became part of the curriculum of public health course in selected universities.

**Figure 1:**
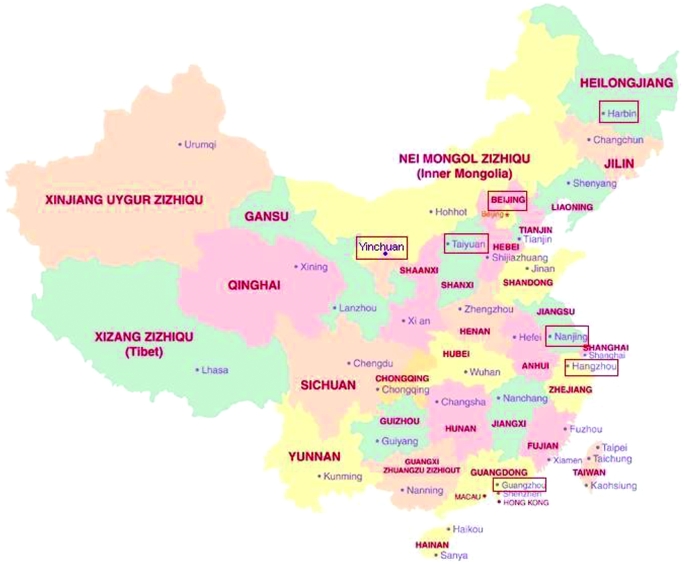
Map showing the seven Chinese universities involved in the project.

Seven Chinese universities were involved (see fig 1): Beijing University, Harbin Medical University, Ningxia Medical University, Guangdong Pharmaceutical College, Shanxi Medical University, Zhejiang University and Nanjing Medical University.

There were 3315 public health students in these institutions and 21.8% (722) of them were in the semester when the tobacco control advocacy training was part of the curriculum. These institutions were selected as they were regionally diverse; they had an existing collaborative network with the primary investigator; and they were committed to introducing the tobacco control advocacy curriculum into their university.

## Implementing the programme

The main activities of the programme are listed below:

1. *Establish project programme parameters:* This involved carrying out an international literature review, interviewing experienced international advocates to identify advocacy strategies suitable for public health graduates working in China and implementing a smoke-free campus advocacy programme.^5^ ^6^
2. *Determine a culturally appropriate tobacco control teaching programme:* This involved interviews with cultural leaders, officials and health experts to determine appropriate strategies, taking into account the current environment for tobacco control in China.
3. *Develop guidelines for project implementation:* Participating universities were involved in this activity, along with experts and officials.
4. *Establish an evaluation programme:* This covered formative evaluation and subsequent refinement of the approach using process evaluation to monitor implementation and evaluation of outcomes.
5. *Training for the programme:* A four-day training workshop for teaching staff of the seven universities, led by national and international experts, was held in Hangzhou in October 2008. The training component in the workshops addressed:
  - the international and Chinese tobacco policies
  - the FCTC and tobacco control policies
  - tobacco control advocacy theories, strategies and methods
  - the plan and implementation of tobacco control advocacy activities
  - evaluation methods for tobacco control advocacy.

The project involved the development of a new organisation in Zhejiang University—the Centre for Tobacco Control Research School of Medicine, which is the only fully tobacco control research organisation in China, and the first tobacco control organisation in any university in China. This infrastructure had the key role in the project implementation and other tobacco control activities that will sustain the partnership and training activities among universities.

## Teaching the new curriculum in the seven universities

A wide range of teaching methodologies was then used by the trained staff to teach the new curriculum in their institutions. These included lectures, problem-based learning, group discussions, role play, debate and case studies. The main aim was to equip students with an understanding of the basic theories, methodology and skills required for promoting tobacco control activities.

The course involved eight contact (classroom) hours and eight non-contact hours. Contact hours included two hours on tobacco epidemiology and the FCTC, four hours on tobacco control advocacy and politics and two hours on in-class practice (development of tobacco control advocacy plans). Two to three teachers implemented the teaching in each of the seven universities. These teachers were then responsible for teaching the public health students. Class sizes ranged from 29 to 115.

The most critical element of the curriculum was that teachers and students were encouraged to establish and implement a smoke-free campus policy in their own universities. This involved a number of key activities including development of themes and slogans for activities to support tobacco control. These played an important and positive part in the programme.

Students were divided into small groups of four to six individuals to prepare a “smoke-free campus advocacy plan” for their own campus. They presented their proposals to an audience which included members of the public, who later voted for the best paper presented in the summing-up workshop.

**Figure 2:**
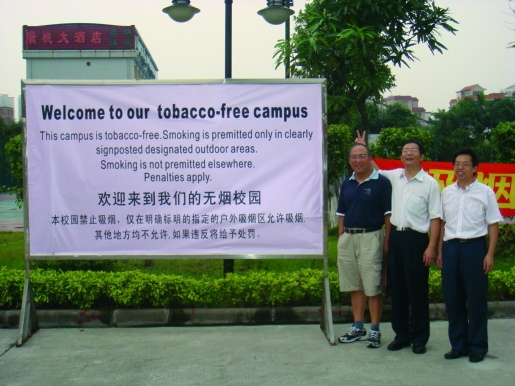
“No-smoking” signage in Guangdong Pharmaceutical College campus.

An advocacy coalition group was created in each university. This included teachers and students of public health, personnel from the local centre for disease control, university administrative personnel, media representatives, non-governmental organisation (NGO) representatives such as the Women’s Union, Student’s Union, Labour Union and members of the public. Their activities included:

- producing signs and banners (see fig 2)
- policy advocacy activities, such as lobbying university leaders and contacting the press
- multiple evaluation activities such as measuring other students’ knowledge of tobacco control issues and monitoring changes in levels of smoking indoors on campus.

## Results

These can be summarised as follows:

1. Tobacco control advocacy training has been ratified by teaching administration departments and has been embedded in the public health curriculum in seven universities in China.
2. A training programme in tobacco control advocacy that can uniquely fit the Chinese culture has been developed.
3. Skilled teaching groups have been organised, which are competent to deliver effective tobacco control advocacy courses to successive cohorts of students in the involved universities.
4. The knowledge and skills of tobacco control advocacy of the public health students increased in the selected universities, as assessed in written assignments.
5. Smoke-free campus policies were developed and implemented in six universities (Beijing University already had a smoke-free policy). The policies recommended no smoking indoors, with designated smoking areas outdoors.

## Discussion

No major obstacles were encountered in implementing the curriculum in participating universities. However there were some issues relating to standardising and supervising the implementation to achieve the target.

The project also highlighted the need for an approach that worked both “top down” and “bottom up” in recognition that policies calling for the authority of government or bureaucracy alone will have limited effect and work best in combination with a “grassroots” style approach.

## Future plans

The general consensus from the university academics involved is that this project has been successful and should be further extended to other medical universities that are similarly committed to introducing a tobacco control curriculum within their universities.^7^ An official (Xiaochao Xu) from the National Health Ministry expressed her affirmative attitude towards the future expansion of the project. She thinks it is urgent that the public health workforce in China develops advocacy capacity for tobacco control. A newspaper of the National Health Ministry (*Health Newspaper*) reported on our project and quoted her as saying “it is urgent to conduct tobacco control advocacy training in our country”.

## Building tobacco control capacity

China is in the midst of sweeping sociological change as the rigid social structure breaks down and new ideology replaces old. This project involves changing social attitudes and norms away from smoking, and this may in turn promote further government support for change as the capacity for building tobacco control increases.
